# Fabry Disease: Integrating Molecular Pathophysiology, Precision Diagnosis, and Artificial Intelligence Toward Precision Medicine

**DOI:** 10.3390/cells15171614

**Published:** 2026-09-04

**Authors:** Giuseppa Biddeci, Gaetano Spinelli, Paolo Colomba, Monia Anania, Giovanni Duro, Francesco Di Blasi

**Affiliations:** Institute for Biomedical Research and Innovation, National Research Council of Italy (IRIB-CNR), Via Ugo La Malfa 153, 90146 Palermo, Italy; giuseppa.biddeci@irib.cnr.it (G.B.); gaetano.spinelli@irib.cnr.it (G.S.); paolo.colomba@irib.cnr.it (P.C.); monia.anania@irib.cnr.it (M.A.); giovanni.duro@irib.cnr.it (G.D.)

**Keywords:** Fabry disease, inflammation, precision diagnosis, artificial intelligence, multi-omics

## Abstract

**Highlights:**

**What are the main findings?**

- Fabry disease involves interconnected pathogenic mechanisms underlying substantial clinical and phenotypic heterogeneity.
- Precision diagnosis requires integration of clinical, genetic, biochemical, imaging, and molecular information.

**What are the implications of the main findings?**

- AI can integrate multidimensional data to support early diagnosis, risk stratification, biomarker discovery, and therapeutic decision-making.
- AI-enabled multimodal approaches may advance Fabry disease toward predictive and personalized management.

**Abstract:**

Fabry disease is a rare X-linked lysosomal storage disorder caused by pathogenic variants in the *GLA* gene, resulting in deficient α-galactosidase A activity and progressive accumulation of globotriaosylceramide (Gb3) and globotriaosylsphingosine (lyso-Gb3). Although lysosomal substrate storage represents the primary molecular defect, accumulating evidence indicates that disease progression is driven by interconnected mechanisms, including chronic inflammation, oxidative stress, endothelial dysfunction, and impaired autophagy, leading to progressive multisystem involvement. The marked clinical heterogeneity of Fabry disease, together with nonspecific early manifestations, frequently delays diagnosis and complicates patient stratification and therapeutic decision-making. While advances in biomarkers, genetic testing, and imaging have improved disease recognition, current diagnostic approaches remain insufficient to fully capture disease complexity. Precision medicine is therefore emerging as a promising strategy through the integration of clinical, molecular, imaging, and multi-omics data. In this context, artificial intelligence (AI) offers novel opportunities for early diagnosis, biomarker discovery, risk stratification, and prediction of therapeutic response. This review provides an integrated overview of the molecular mechanisms, inflammatory pathways, clinical manifestations, and precision diagnostic strategies underlying Fabry disease, highlighting how AI-driven approaches may accelerate the transition toward more accurate, personalized, and predictive disease management.

## 1. Introduction

Fabry disease (FD; OMIM #301500), also known as Anderson–Fabry disease, is a rare X-linked lysosomal storage disorder caused by pathogenic variants in the *GLA* gene, located on chromosome Xq22.1, which encodes the lysosomal enzyme α-galactosidase A (α-GalA). Reduced or absent α-GalA activity impairs the degradation of glycosphingolipids, resulting primarily in lysosomal accumulation of globotriaosylceramide (Gb3) and increased levels of its soluble deacylated derivative, globotriaosylsphingosine (lyso-Gb3), a bioactive metabolite and established biomarker of disease burden [1,2,3]. Progressive substrate accumulation affects multiple cell types, including endothelial cells, cardiomyocytes, podocytes, neurons, and immune cells, ultimately leading to multisystemic tissue injury. The clinical spectrum of FD is highly heterogeneous, ranging from early-onset multisystemic disease to later-onset phenotypes with predominant cardiac or renal involvement [4,5]. The estimated incidence of classic Fabry disease has been reported at approximately 1:40,000–1:60,000 males, although the increasing recognition of later-onset phenotypes suggests that the overall frequency of disease may be substantially higher [6]. Major clinical manifestations include neuropathic pain, gastrointestinal disturbances, angiokeratomas, renal impairment, left ventricular hypertrophy, arrhythmias, and cerebrovascular complications [7,8,9,10]. Phenotypic variability is particularly evident among heterozygous females in whom random and skewed X-chromosome inactivation (XCI) can result in variable proportions of cells expressing the mutant and wild-type *GLA* alleles. Phenotypic variability is also observed in individuals carrying variants associated with residual α-GalA activity [11,12,13]. Increasing evidence indicates that substrate accumulation alone cannot fully explain this heterogeneity. Instead, FD progression involves interconnected secondary mechanisms, including lysosomal and autophagic dysfunction, oxidative stress, chronic inflammation, endothelial dysfunction, immune activation, and progressive fibrosis [14,15,16,17,18,19,20,21]. Despite advances in disease-specific treatments, including enzyme replacement therapy (ERT) and pharmacological chaperones, clinical outcomes remain influenced by the timing of treatment initiation, baseline organ damage, and individual molecular and clinical characteristics [22]. Delayed diagnosis, incomplete genotype–phenotype correlations, and the limited ability of conventional biomarkers to capture disease heterogeneity remain important challenges [4,23,24,25,26]. These limitations reflect the broader biological and clinical complexity of FD, which poses significant challenges for traditional diagnostic and therapeutic approaches. The increasing availability of genetic, biochemical, imaging, and longitudinal clinical data has created new opportunities for a more comprehensive understanding of disease mechanisms but also requires advanced analytical tools capable of integrating multidimensional datasets. In this context, precision medicine approaches aimed at tailoring diagnosis and treatment based on individual molecular and clinical profiles are becoming increasingly relevant. Artificial intelligence (AI) has emerged as a powerful tool to address these challenges by integrating and analyzing complex, multimodal datasets. In Fabry disease, AI and machine learning approaches can facilitate earlier and more accurate diagnosis, patient stratification, prediction of disease progression, and therapeutic decision-making. Their applications increasingly encompass genetic data interpretation, biomarker discovery, imaging analysis, and prediction of treatment response, including to enzyme replacement therapy, pharmacological chaperones, and emerging strategies such as substrate reduction and gene-based therapies [27,28,29]. The present review provides an integrated overview of Fabry disease, focusing on the molecular and cellular mechanisms underlying disease progression, the challenges of precise diagnosis and patient stratification, and the emerging role of AI in precision medicine.

## 2. Molecular and Cellular Pathophysiology of Fabry Disease

**Figure 1:**
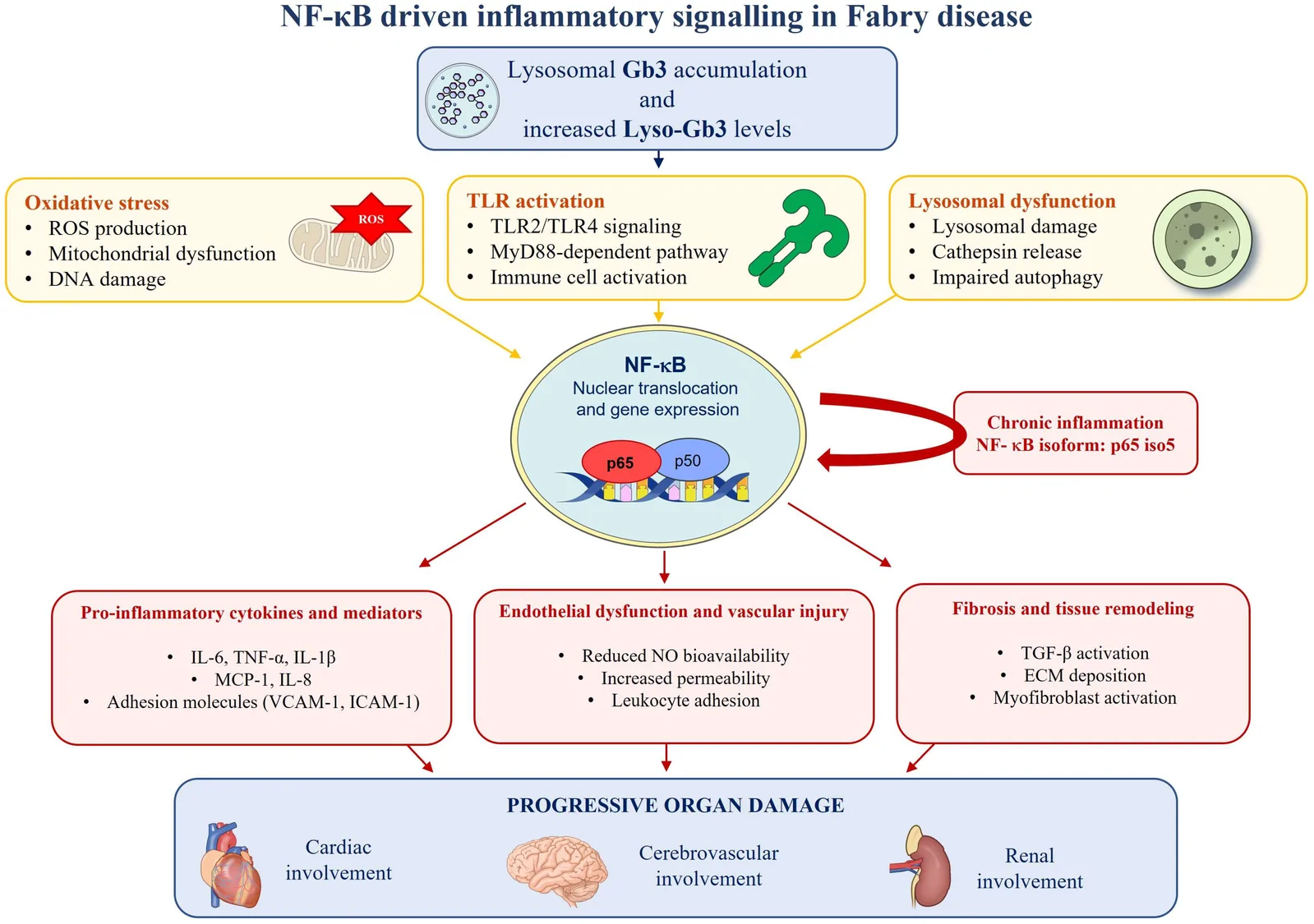
NF-κB-driven inflammatory signaling in Fabry disease. Lysosomal globotriaosylceramide (Gb3) storage and elevated globotriaosylsphingosine (lyso-Gb3) levels trigger reactive oxygen species (ROS) production, Toll-like receptor (TLR2/TLR4) activation via the myeloid differentiation primary response 88 (MyD88)-dependent pathway, and lysosomal dysfunction. These upstream events converge on nuclear factor kappa B (NF-κB) nuclear translocation and gene expression. Activated NF-κB signaling promotes the transcription of pro-inflammatory cytokines and chemokines (including interleukin-6, IL-6; tumor necrosis factor-alpha, TNF-α; interleukin-1 beta, IL-1β; monocyte chemoattractant protein-1, MCP-1; and interleukin-8, IL-8) and cell adhesion molecules (vascular cell adhesion molecule 1, VCAM-1; intercellular adhesion molecule 1, ICAM-1). Concurrently, NF-κB induces endothelial dysfunction (characterized by reduced nitric oxide (NO) bioavailability) and profibrotic pathways involving transforming growth factor-beta (TGF-β) activation and extracellular matrix (ECM) deposition, ultimately driving progressive multi-organ damage. Reduced expression of p65 iso5 may contribute to dysregulated NF-κB signaling and persistent inflammatory activation in Fabry disease. Positive feedback loops further sustain inflammatory activation and disease progression.

Fabry disease results from pathogenic variants in the *GLA* gene that impair the structure, stability, catalytic activity, or intracellular processing of α-galactosidase A (α-GalA), a lysosomal hydrolase responsible for the degradation of terminal α-galactosyl residues from Gb3 and related glycosphingolipids [30,31]. When α-GalA activity is deficient or absent, Gb3 progressively accumulates within lysosomes, disrupting lysosomal function and initiating a cascade of downstream pathogenic events that ultimately impair cellular homeostasis and tissue integrity [15,32]. More than 1000 *GLA* variants have been identified, including missense and nonsense variants, splice-site alterations, small insertions and deletions, and, less frequently, larger structural rearrangements [33]. These variants can affect enzyme catalytic activity, protein folding and stability, intracellular trafficking, or substrate recognition, resulting in variable degrees of α-GalA deficiency [34,35,36]. The resulting reduction or loss of α-GalA activity represents the primary molecular defect and determines, at least in part, the extent and timing of glycosphingolipid accumulation. Individuals with minimal or absent residual enzyme activity generally develop the classic phenotype, characterized by early and progressive multisystemic involvement, whereas variants retaining partial enzymatic activity are frequently associated with later-onset disease, often with predominant cardiac or renal manifestations [37,38,39,40]. However, the relationship between *GLA* genotype, residual enzymatic activity, and clinical phenotype is not strictly linear. In heterozygous females, cellular mosaicism resulting from X-chromosome inactivation further contributes to phenotypic variability, while modifier genes, epigenetic mechanisms, cellular stress responses, and environmental factors may additionally influence disease expression [11,41,42,43,44,45]. The deacylated and soluble derivative lyso-Gb3 also increases in plasma and tissues and may exert biological effects beyond its role as a disease biomarker, promoting cellular stress, inflammatory signaling, smooth muscle cell proliferation, and profibrotic responses [14,46]. Lysosomal dysfunction is accompanied by impaired autophagic flux and reduced clearance of damaged proteins and organelles, contributing to mitochondrial dysfunction and increased production of reactive oxygen species (ROS) [47,48,49,50]. Elevated oxidative stress has been demonstrated in endothelial cells, cardiomyocytes, and renal cells derived from Fabry patients, leading to lipid peroxidation, DNA damage, and mitochondrial dysfunction [51,52]. These alterations may promote the activation of inflammatory pathways, including nuclear factor kappa B (NF-κB), Toll-like receptor (TLR) signaling, and inflammasome-related mechanisms, thereby contributing to the production of pro-inflammatory cytokines and chemokines, such as TNF-α, IL-6, IL-1β, and IL-8, as well as to altered activation of circulating immune cells [14,18,20,52]. NF-κB signaling may therefore represent an important molecular link between glycosphingolipid accumulation, cellular stress, inflammation, endothelial dysfunction, and tissue remodeling in FD, providing further evidence that dysregulated NF-κB-related pathways may contribute to disease progression [14,15]. These interconnected mechanisms are summarized in Figure 1.

**Figure 2:**
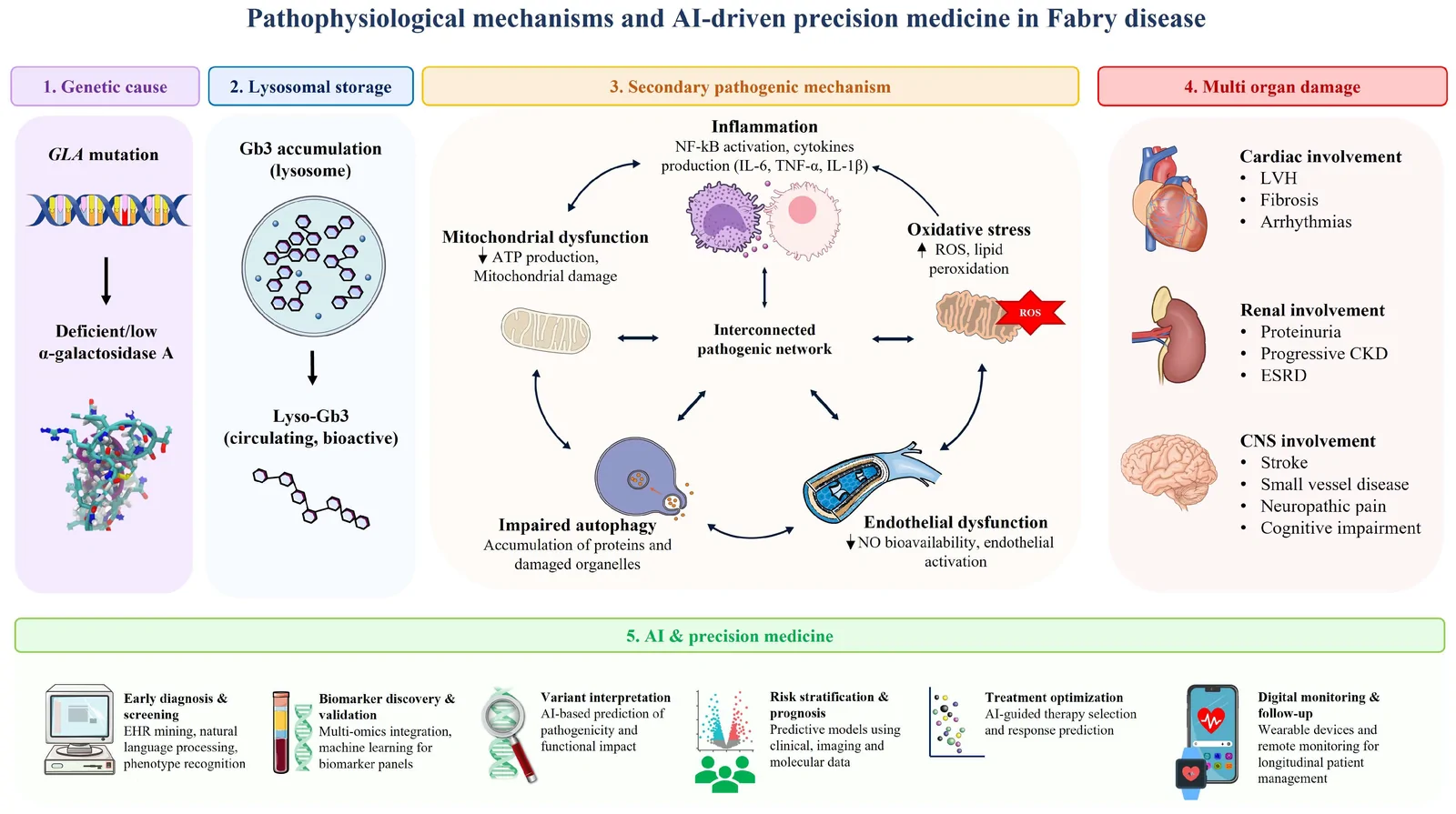
**Integrated pathophysiological mechanisms and AI-driven precision medicine in Fabry disease.** Fabry disease is caused by pathogenic variants in the *GLA* gene, resulting in α-galactosidase A deficiency, progressive lysosomal storage of globotriaosylceramide (Gb3), and generation of the bioactive metabolite globotriaosylsphingosine (lyso-Gb3). Together, Gb3 storage and circulating lyso-Gb3 accumulation promote chronic inflammation, oxidative stress, endothelial dysfunction, impaired autophagy, and mitochondrial dysfunction, ultimately leading to progressive multi-organ damage involving the heart (e.g., left ventricular hypertrophy, LVH), kidneys (e.g., chronic kidney disease, CKD; end-stage renal disease, ESRD), and nervous system. Artificial intelligence (AI) approaches enable the integration of electronic health record (EHR) mining alongside multidimensional genetic, molecular, imaging, and clinical data to support early diagnosis, biomarker discovery, risk stratification, and personalized therapeutic optimization.

Endothelial dysfunction represents another important component of this interconnected pathogenic network. Accumulation of Gb3 in endothelial cells, together with oxidative and inflammatory stress, can impair nitric oxide (NO) bioavailability and disrupt vascular homeostasis, resulting in impaired vasodilation, increased vascular stiffness, and a pro-thrombotic and pro-inflammatory vascular environment. Endothelial dysfunction may consequently contribute to microvascular ischemia and tissue injury underlying cardiac, renal, and neurological complications. Glycosphingolipid accumulation also interferes with autophagy, lysosomal function, and intracellular signaling pathways, promoting apoptosis, cellular senescence, and fibrosis. Activation of profibrotic pathways, including transforming growth factor beta (TGF-β) signaling, further contributes to extracellular matrix deposition and irreversible tissue remodeling [5,19,21,29,51]. Importantly, the extent of substrate accumulation does not always correlate directly with clinical severity, and individuals carrying the same *GLA* variant may develop markedly different phenotypes [19,20,21,43,44,45]. This biological complexity provides an important rationale for integrated approaches combining genetic, biochemical, molecular, imaging, and clinical information to improve disease characterization and patient stratification (Figure 2).

## 3. Clinical Spectrum and Phenotypic Heterogeneity

**Figure 3:**
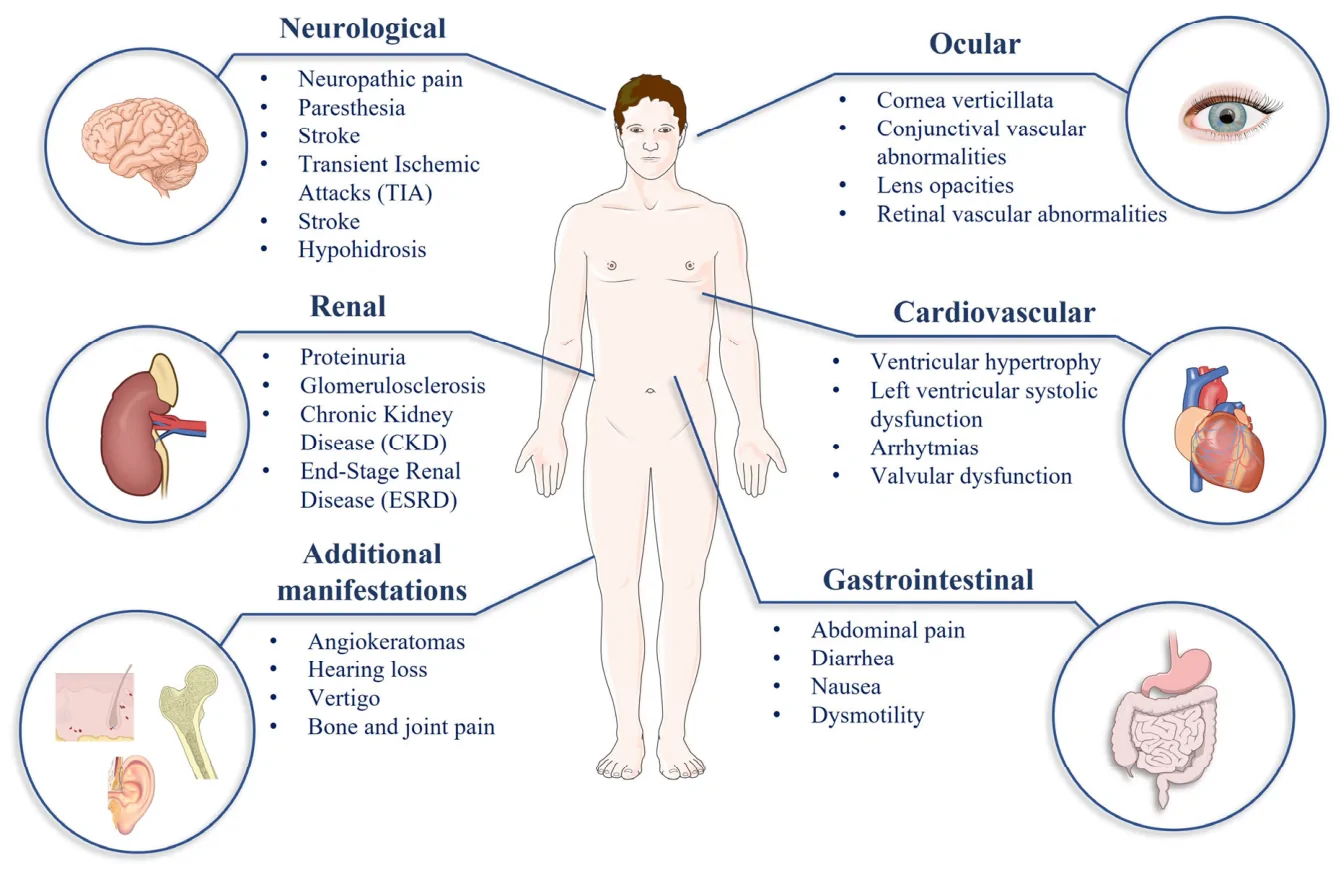
Multisystemic clinical manifestations of Fabry disease. Fabry disease is characterized by a broad and heterogeneous clinical spectrum involving multiple organ systems. Neurological manifestations include neuropathic pain, stroke, and transient ischemic attacks. Ophthalmological findings include cornea verticillata and retinal vascular abnormalities. Renal involvement typically presents with proteinuria and may progress to chronic kidney disease and end-stage renal disease. Cardiac manifestations include left ventricular hypertrophy, arrhythmias, and valvular dysfunction. Gastrointestinal symptoms comprise abdominal pain, diarrhea, nausea, and dysmotility. Additional features include angiokeratomas, hypohidrosis, hearing loss, and musculoskeletal pain. The variability in organ involvement and disease severity reflects the underlying phenotypic heterogeneity of Fabry disease.

Fabry disease is characterized by a broad and highly heterogeneous clinical spectrum, reflecting the complex interplay between genetic, biochemical, and environmental factors. Disease manifestations can vary widely in terms of age of onset, severity, and organ involvement, ranging from the classic early-onset phenotype to later-onset variants predominantly affecting the heart or kidneys. This remarkable heterogeneity reflects the widespread accumulation of glycosphingolipids across multiple cell types and tissues, leading to progressive multisystem involvement (Figure 3). Rather than representing isolated organ-specific manifestations, the clinical phenotype of Fabry disease arises from common pathogenic mechanisms involving lysosomal dysfunction, chronic inflammation, endothelial injury, oxidative stress, impaired autophagy, and progressive fibrosis. The interaction of these processes drives cellular dysfunction across multiple organs while contributing to the marked variability observed among affected individuals. The cardiovascular system is one of the primary targets of disease involvement and represents a major determinant of morbidity and mortality. Glycosphingolipid accumulation within cardiomyocytes, endothelial cells, and cardiac fibroblasts promotes hypertrophic remodeling, microvascular dysfunction, and fibrotic progression, ultimately leading to left ventricular hypertrophy, myocardial fibrosis, conduction abnormalities, arrhythmias, and heart failure [39,53,54]. Likewise, renal involvement results from progressive glycolipid deposition in podocytes, mesangial cells, glomerular endothelial cells, and tubular epithelial cells, causing proteinuria, chronic kidney disease, and, in untreated patients, end-stage renal disease. Podocyte injury is now recognized as one of the earliest pathological events and closely correlates with disease progression [55,56,57,58]. Neurological manifestations involve both the peripheral and central nervous systems. Small-fiber neuropathy is responsible for the characteristic acroparesthesias frequently observed during childhood, whereas cerebrovascular complications, including transient ischemic attacks and stroke, reflect endothelial dysfunction, chronic neuroinflammation, and microvascular injury. Increasing evidence indicates that sustained NF-κB activation contributes to neuronal damage and vascular pathology, linking lysosomal dysfunction with progressive neurological impairment [18,59,60,61,62,63,64,65]. Additional manifestations include hearing loss, tinnitus, vertigo, angiokeratomas, hypohidrosis, and gastrointestinal symptoms, which arise from autonomic dysfunction, vascular alterations, and glycosphingolipid accumulation within affected tissues [59,64,66,67,68,69]. The principal molecular mechanisms, clinical manifestations, and representative biomarkers associated with the major organ systems involved in Fabry disease are summarized in Table 1. The extent and pattern of organ involvement differ markedly among patients and are influenced by residual α-GalA activity, sex, genetic background, and additional disease-modifying factors. While hemizygous males with little or no residual enzymatic activity typically develop the classic severe phenotype, heterozygous females exhibit highly variable clinical expression because of random X-chromosome inactivation (XCI), resulting in a spectrum ranging from asymptomatic individuals to severe multiorgan disease [70,71]. Importantly, significant organ damage may precede overt clinical manifestations, emphasizing the need for early diagnosis and longitudinal monitoring. The substantial phenotypic variability observed in Fabry disease remains one of the major challenges for patient stratification and individualized clinical management. Conventional genotype–phenotype correlations alone are often insufficient to accurately predict disease trajectory, underscoring the need for integrative approaches combining molecular, biochemical, imaging, and clinical data. This complexity provides a strong rationale for the application of advanced computational tools and artificial intelligence-based models, which are increasingly being investigated to improve patient classification, risk prediction, and personalized therapeutic decision-making [4,27,72].

## 4. Early and Precision Diagnosis in Fabry Disease

**Figure 4:**
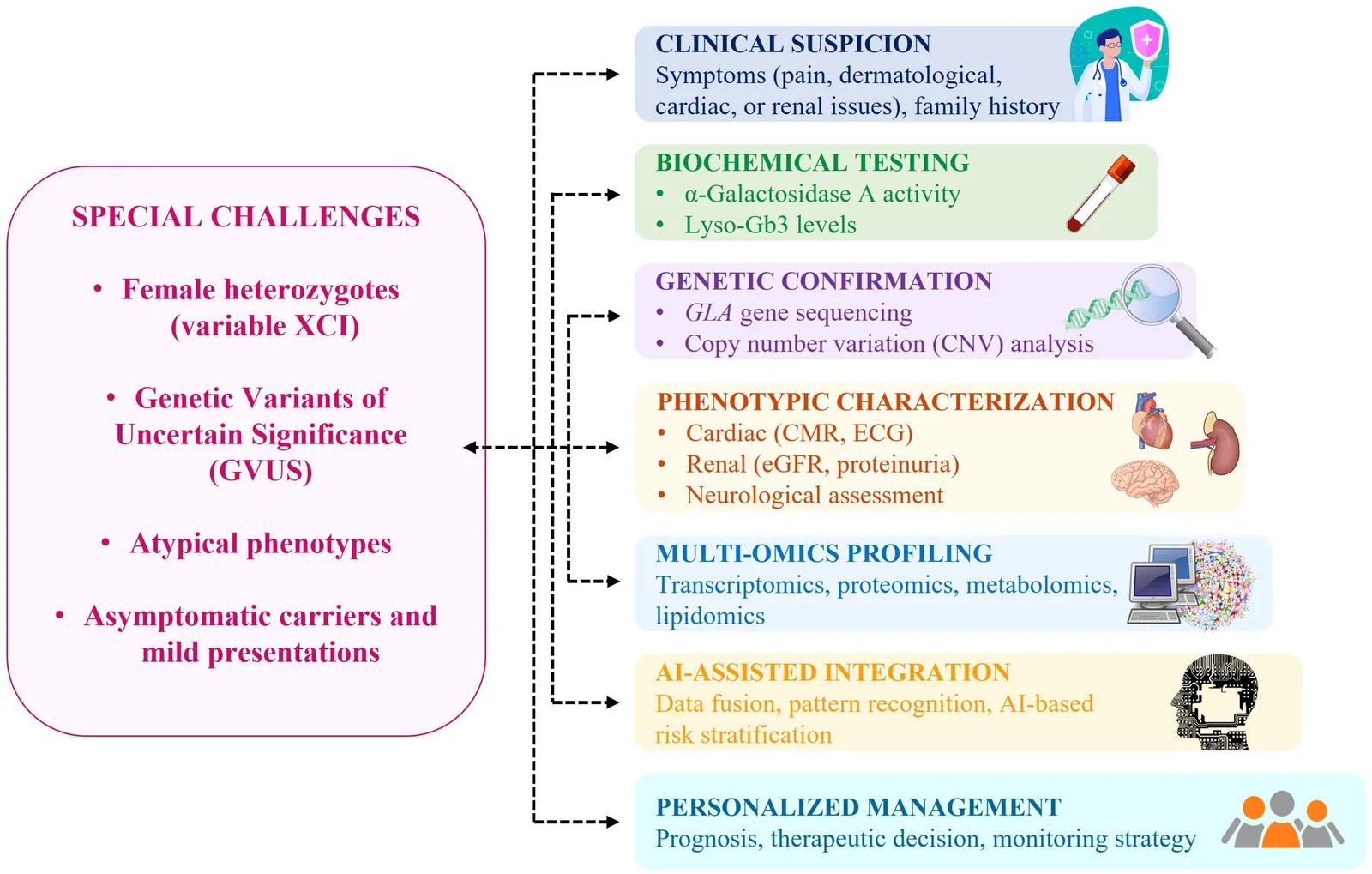
Multidimensional diagnostic framework for early and precision diagnosis in Fabry disease. Fabry disease diagnosis requires an integrated approach combining clinical evaluation, biochemical testing (α-galactosidase A activity and lyso-Gb3), genetic analysis (*GLA* sequencing with copy number variation (CNV) analysis when indicated), and comprehensive phenotypic characterization. Advanced imaging techniques, including cardiac magnetic resonance (CMR), enable early detection of organ involvement. Multi-omics approaches, including transcriptomics, proteomics, metabolomics, and lipidomics, provide complementary molecular insights, while artificial intelligence (AI) integrates clinical, biochemical, genetic, imaging, and multi-omics data to improve early diagnosis, variant interpretation, patient stratification, and personalized disease management.

Early and accurate diagnosis of Fabry disease is essential, as irreversible organ damage may occur before clinically evident symptoms. However, diagnostic delay remains a major challenge, largely due to disease rarity, heterogeneous clinical presentation, and overlap with more common disorders. This is particularly relevant in later-onset phenotypes and in heterozygous females, in whom clinical manifestations may be mild or atypical. These challenges have promoted the implementation of systematic screening strategies, including newborn screening and family cascade screening, which facilitate the identification of affected or at-risk individuals before the onset of clinically evident disease [74,75,76,77]. Biochemical testing represents the first-line approach in males. Measurement of α-galactosidase A (α-GalA) enzymatic activity in plasma, leukocytes, or dried blood spots provides a reliable and highly specific diagnostic marker in hemizygous males, in whom markedly reduced or absent enzyme activity strongly supports the diagnosis. However, in heterozygous females, enzymatic activity may fall within the normal range due to random XCI, limiting the sensitivity of this approach. Consequently, molecular genetic analysis is required for definitive diagnosis in females and for confirmation in all patients [78]. Genetic testing, based on sequencing of the *GLA* gene, constitutes the diagnostic gold standard. It enables the identification of pathogenic variants, supports genotype–phenotype correlations, and provides the basis for genetic counseling and family screening [79]. The increasing identification of genetic variants of uncertain significance (GVUS), however, represents a major challenge and requires the integration of biochemical, genetic, functional, and clinical evidence for appropriate interpretation [80,81]. Among biochemical biomarkers, lyso-Gb3 has emerged as the most informative and widely used marker in FD. Its plasma levels are markedly elevated in males with classic disease and are also increased in many affected females. Additional biomarkers, including inflammatory mediators, endothelial dysfunction markers, and acute-phase proteins, are currently under investigation, although none have yet been validated for routine clinical use [26]. Because no single biomarker adequately captures the biological and clinical complexity of Fabry disease, increasing attention has shifted toward multimodal diagnostic strategies that integrate biochemical biomarkers, advanced imaging, genetic information, and omics-derived data to achieve a more comprehensive characterization of disease status. Advanced imaging and multi-omics approaches provide complementary information on organ involvement and disease biology. Cardiac magnetic resonance imaging (MRI), particularly native T1 mapping, can detect early myocardial involvement and may identify cardiac abnormalities before overt hypertrophy develops [82]. Renal imaging and histological assessment can similarly provide information on early structural alterations [83]. Emerging omics approaches, including proteomics [84,85], metabolomics [52,86], and lipidomics [87,88], are expanding the diagnostic landscape of Fabry disease by identifying disease-specific molecular signatures associated with metabolic dysregulation, inflammation, oxidative stress, and lysosomal dysfunction. The integration of these complementary data layers with clinical and biochemical information represents a key step toward precision diagnosis, enabling more comprehensive disease characterization and patient stratification (Figure 4).

The principal components of this framework, together with their strengths, limitations, and potential AI applications, are summarized in Table 2.

## 5. Artificial Intelligence in Fabry Disease Management

The growing availability of multidimensional clinical, genetic, biochemical, imaging, and multi-omics datasets has exceeded the analytical capacity of conventional statistical approaches, creating a strong rationale for artificial intelligence (AI)-based models capable of integrating heterogeneous sources of information into clinically meaningful insights. In this context, AI is increasingly recognized as an enabling technology for precision diagnosis and personalized disease management in Fabry disease. Machine learning (ML) algorithms can integrate multimodal datasets to identify disease-specific patterns that are often not readily detectable using conventional analytical methods, enabling a more comprehensive characterization of disease heterogeneity [27]. AI-based tools have already demonstrated promising performance across several aspects of Fabry disease diagnosis. Predictive models applied to electronic health records (EHRs) have achieved high discriminative accuracy in identifying individuals with clinical patterns suggestive of Fabry disease, enabling targeted confirmatory testing [92,93]. Likewise, deep learning approaches applied to advanced imaging, including cardiac magnetic resonance (CMR), and to urinary sediment analysis have shown encouraging results for the identification of disease-specific features, highlighting their potential for non-invasive screening and early diagnosis. Natural language processing (NLP) techniques further expand these capabilities by extracting clinically relevant information from unstructured medical records and generating risk scores that support the early identification of suspected cases [94]. Beyond case detection, AI also holds considerable promise for improving the interpretation of *GLA* variants, particularly variants of uncertain significance (GVUS), through the integration of functional, structural, biochemical, and population-level data [89,90]. More broadly, AI has the potential to combine clinical evaluation, biochemical biomarkers, genetic findings, imaging, and multi-omics information into unified predictive models that improve patient stratification, estimate disease severity, anticipate clinical progression, and ultimately support individualized therapeutic decision-making. Despite these promising advances, several challenges continue to limit the widespread implementation of AI in clinical practice. Most available studies rely on relatively small patient cohorts because of the rarity of Fabry disease, increasing the risk of model overfitting and limiting generalizability. Furthermore, the lack of standardized datasets, external validation across independent populations, and harmonized data collection protocols remains a major barrier to clinical translation. Additional challenges include model interpretability, regulatory requirements, data privacy, and integration into routine clinical workflows. Addressing these limitations will be essential to ensure that AI systems become robust, transparent, and clinically reliable tools for precision medicine. As these challenges are progressively overcome, the integration of AI with multi-omics, advanced imaging, and longitudinal clinical datasets is expected to enable truly personalized diagnostic and therapeutic pathways, facilitating earlier disease detection, more accurate risk stratification, optimized treatment selection, and long-term disease monitoring. Ultimately, AI should not be regarded as a replacement for conventional diagnostic approaches but rather as a complementary technology capable of integrating heterogeneous biomedical information into clinically actionable knowledge, thereby advancing precision medicine in Fabry disease.

### 5.1. AI for Precision Diagnosis and Risk Stratification

One of the most clinically impactful applications of AI in Fabry disease lies in early detection and risk stratification. Diagnostic delay remains a major unmet need, particularly in patients with atypical or late-onset phenotypes and in heterozygous females. Machine learning models applied to electronic health records (EHRs) have demonstrated the ability to identify high-risk individuals by capturing subtle combinations of clinical features, significantly improving case-finding efficiency compared to traditional approaches [93]. Recent advances further extend this concept through hybrid approaches combining expert knowledge and AI. The integration of clinician-driven rules with machine learning models has been shown to enhance the performance of symptom checkers for rare disease identification, improving sensitivity and supporting earlier suspicion in clinical practice [29]. Importantly, sex-specific differences are emerging as a critical factor in AI-based diagnostics. Data mining approaches have demonstrated that incorporating gender-related variability significantly improves diagnostic accuracy, particularly in females [95]. These findings highlight the importance of developing bias-aware AI systems that account for biological and clinical heterogeneity. Deep learning is also transforming non-invasive and histological diagnostics. Automated urinary sediment analysis using convolutional neural networks has shown high accuracy in detecting Fabry-specific features [96]. In parallel, the ZEBRA (ZEbra Bodies Recognition by Artificial Intelligence tool) has demonstrated the ability to recognize zebra bodies in renal biopsy specimens, improving diagnostic standardization and reducing observer variability [97]. AI applications extend beyond renal pathology. Retinal microvasculature analysis using deep learning has revealed early systemic vascular alterations associated with cerebral small vessel disease [98]. Similarly, neuroimaging approaches based on the brain-age paradigm have demonstrated accelerated brain aging in Fabry patients, providing quantitative markers of subclinical neurological involvement [99]. At the molecular level, AI-driven integration of multi-omics datasets enables the discovery of novel biomarkers. Transcriptomic and promoter-level analyses combined with machine learning have identified candidate predictors of cardiac complications, further refining risk stratification beyond traditional genotype–phenotype correlations [100]. Collectively, these advances indicate that AI is shifting Fabry disease management from organ-specific and descriptive approaches toward integrated, systems-level models capable of supporting predictive, preventive, and personalized medicine.

### 5.2. AI-Driven Therapeutic Optimization

**Figure 5:**
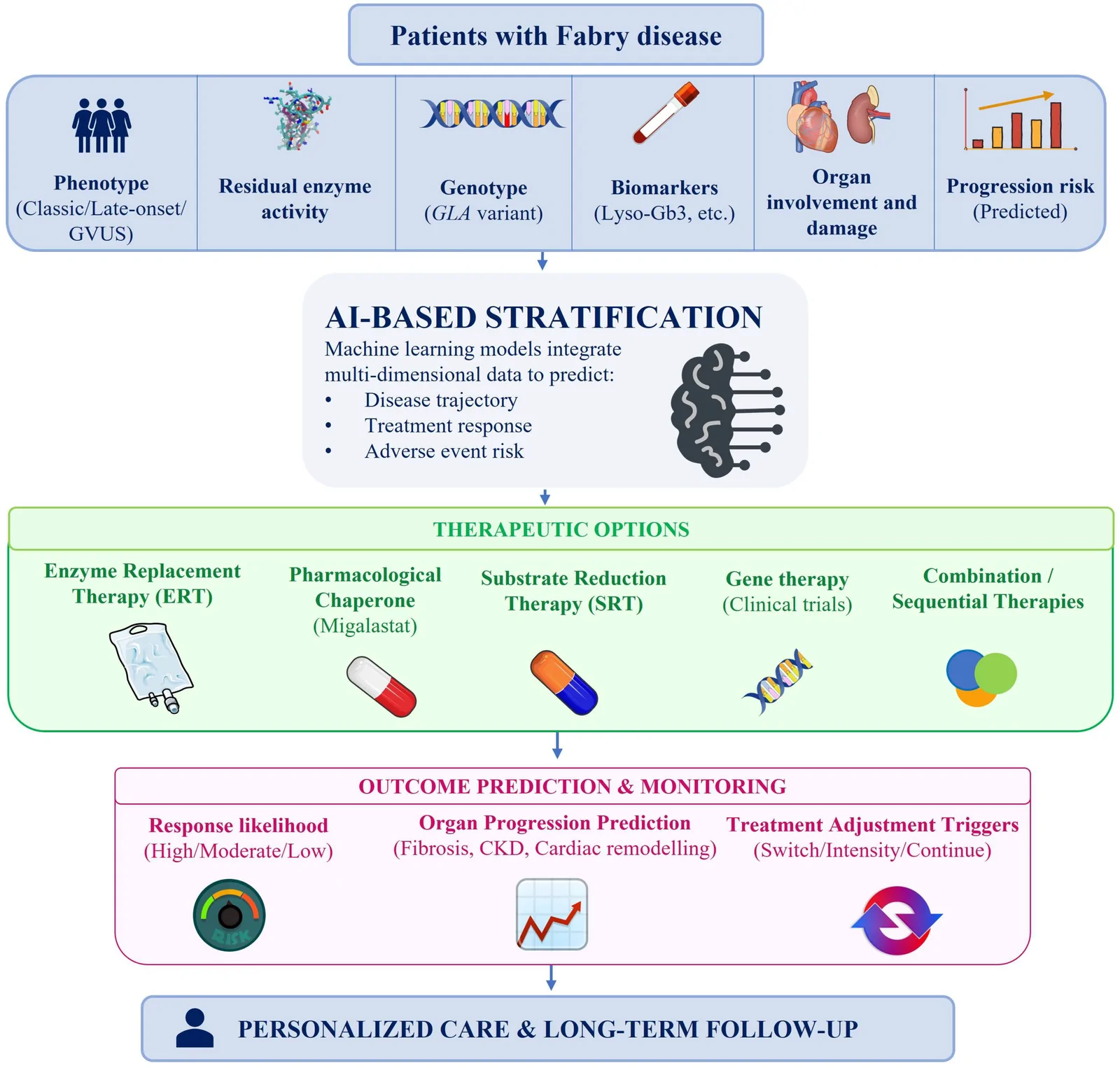
AI-guided therapeutic decision-making in Fabry disease. Therapeutic management of Fabry disease includes enzyme replacement therapy (ERT), pharmacological chaperones, substrate reduction therapy (SRT), and emerging gene therapies. Artificial intelligence can integrate clinical, genetic, biochemical, imaging, and longitudinal data to support treatment-response prediction, patient stratification, therapy selection, and monitoring. This framework illustrates the potential of AI to contribute to personalized treatment strategies aimed at optimizing therapeutic timing and preventing irreversible organ damage.

Beyond improving diagnosis and risk stratification, AI is increasingly being explored to support individualized therapeutic decision-making. Response to Fabry-specific therapies, including enzyme replacement therapy (ERT) and pharmacological chaperones, varies substantially among patients and is influenced by disease stage, genotype, residual enzyme activity, and the extent of established organ damage [101,102]. In this context, AI may help integrate these variables to support more individualized treatment decisions. This emerging paradigm is summarized in an AI-guided therapeutic decision framework integrating molecular, clinical, and longitudinal patient data to support individualized treatment selection (Figure 5). Machine learning approaches are increasingly being applied in nephrology to support treatment-response prediction and therapeutic decision-making, illustrating a potential avenue for similar applications in Fabry disease [103]. This is particularly relevant given that early treatment initiation is essential to prevent irreversible organ damage, while delayed intervention is associated with poorer prognosis. In Fabry disease, AI-based approaches have also been explored for biomarker discovery and prognostic modeling. The integration of clinical and molecular data may enable the identification of signatures associated with disease progression and therapeutic response [27], while AI-assisted histological analysis has recently enabled quantitative assessment of podocyte involvement through the ZEBRA score, with potential applications in the evaluation of Fabry nephropathy [97]. These approaches may ultimately help identify patients at higher risk of progression and refine treatment selection beyond conventional genotype–phenotype correlations. Cardiovascular assessment represents another promising application. Artificial intelligence-enhanced electrocardiography (AI-ECG) has recently been investigated for the detection of cardiac involvement in Fabry disease, demonstrating the potential of ECG-derived features to support early identification and risk assessment of cardiac disease [104]. Such approaches could complement conventional cardiac evaluation and contribute to longitudinal monitoring and treatment optimization. Beyond clinical decision-making, AI is also being increasingly applied to drug discovery and development through deep learning-based screening and computational modeling, an approach particularly relevant to rare diseases with limited therapeutic pipelines [103]. Similarly, AI-based strategies may support the development and optimization of advanced therapies by helping predict treatment efficacy, immune responses, and other determinants of long-term therapeutic outcomes. Collectively, these applications highlight the potential of AI to move Fabry disease management toward a more predictive and individualized therapeutic paradigm.

### 5.3. AI in Clinical Monitoring, Digital Health, and Connected Care

Given the progressive nature of Fabry disease, continuous monitoring is essential. AI is playing an increasingly important role in continuous disease monitoring and connected care models. Digital health technologies, including wearable devices and remote monitoring systems, generate continuous data streams that can be analyzed by AI to detect early signs of disease progression or therapeutic failure [105]. These approaches support the development of connected care ecosystems that integrate clinical data, patient-reported outcomes, and real-world evidence into unified platforms, enabling more proactive and personalized disease management. In nephrology, AI tools are particularly relevant given the central role of renal involvement in Fabry disease. Computational models can support early detection of kidney damage and predict disease progression through the integration of laboratory, imaging, and histological data [103]. AI-powered clinical decision support systems (CDSS) further enhance clinical workflows by providing real-time, personalized recommendations based on both individual patient data and large-scale datasets [27]. Importantly, these technologies contribute to a shift toward predictive, preventive, and participatory medicine, in which patients actively contribute to disease monitoring through digital tools. However, the real-world integration of these technologies into routine clinical practice remains limited.

## 6. Challenges, Bias, and Future Perspectives

**Figure 6:**
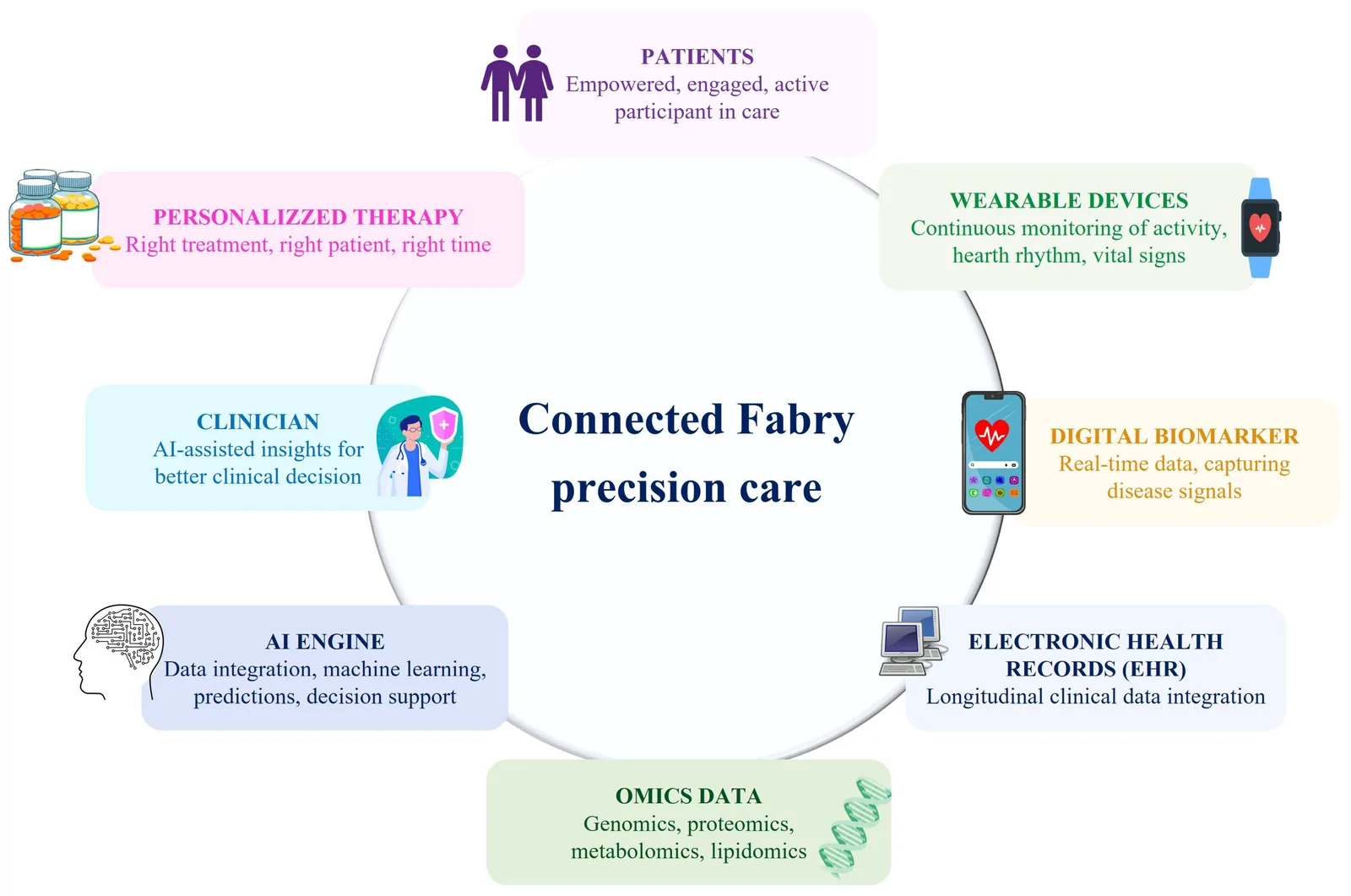
AI-enabled connected care ecosystem in Fabry disease. A future-oriented model of Fabry disease management integrating digital health technologies, wearable devices, and real-world data with clinical, molecular, and imaging information. Artificial intelligence analyzes continuous data streams to enable early detection of disease progression, real-time monitoring, and personalized clinical decision support. This connected-care approach supports predictive, preventive, and participatory medicine.

Despite significant advances in the understanding and treatment of Fabry disease, several challenges continue to limit both therapeutic efficacy and the clinical implementation of emerging technologies such as artificial intelligence (AI). Current disease-specific therapies, including enzyme replacement therapy (ERT) and pharmacological chaperones, are often unable to fully prevent disease progression, particularly when treatment is initiated at advanced stages. Irreversible organ damage, especially fibrosis affecting cardiac and renal tissues, remains only partially responsive to currently available therapies. These limitations underscore the critical importance of early diagnosis and timely intervention. At the same time, novel therapeutic strategies, including gene therapy, mRNA-based approaches, and substrate reduction therapies, offer considerable promise but still raise important concerns regarding long-term efficacy, safety, immunogenicity, and durability of response [101]. Their optimization will require a deeper understanding of disease biology and more precise patient stratification. The marked clinical heterogeneity of Fabry disease further complicates diagnosis, prognostic assessment, and therapeutic decision-making. Incomplete genotype–phenotype correlations, together with the high prevalence of variants of uncertain significance, continue to represent major diagnostic and prognostic challenges, particularly in female patients [95]. These complexities are compounded by the intrinsic limitations of rare diseases, including small and heterogeneous patient cohorts, fragmented datasets, and variability across healthcare systems. From a research perspective, there is therefore a pressing need for large-scale, longitudinal, and multicentric studies integrating clinical, molecular, and imaging data. The development of standardized registries and biobanks will be essential to support robust biomarker discovery and validation. In this context, innovative trial designs, including adaptive and platform trials, may help address the methodological limitations associated with small patient populations. Artificial intelligence and big data integration represent promising tools to overcome many of these challenges, with potential applications in diagnosis, risk stratification, biomarker discovery, and personalized therapeutic decision-making. However, several barriers still limit widespread clinical implementation. Data scarcity and heterogeneity increase the risk of overfitting and reduce model generalizability across different populations and healthcare settings, making external validation in independent cohorts essential [27]. Emerging approaches such as federated learning, which enable collaborative model development without sharing sensitive patient data, may help mitigate data fragmentation and improve scalability. Another major limitation concerns model transparency and interpretability. Many AI systems, particularly deep learning models, function as “black boxes”, limiting clinician trust and adoption. Consequently, the development of explainable AI (XAI) approaches, including feature-attribution methods and model-agnostic interpretation tools, is becoming increasingly important to ensure that model outputs can be understood, validated, and effectively integrated into clinical decision-making [106,107]. Ethical, regulatory, and equity-related considerations also remain central issues. Sex-related differences significantly influence disease presentation and progression and must therefore be adequately incorporated into AI models to avoid diagnostic disparities, especially among female patients [95,106]. More broadly, issues related to data privacy, algorithmic bias, transparency, and equitable access to AI-driven technologies require careful attention [105]. In parallel, the implementation of AI tools in clinical practice must comply with evolving regulatory frameworks and rigorous standards for validation, reproducibility, and safety [108]. Despite encouraging results, most AI applications in Fabry disease remain at the proof-of-concept stage, with limited integration into real-world clinical workflows. Furthermore, the cost-effectiveness of AI-driven strategies in rare diseases has not yet been fully established and will likely represent a critical determinant for large-scale adoption. Looking forward, the future of Fabry disease management will likely depend on the convergence of precision medicine, advanced therapeutics, digital health technologies, and computational approaches. Early diagnosis through integrated screening strategies, combined with personalized therapeutic interventions guided by AI, has the potential to significantly improve patient outcomes and quality of life. The development of multimodal and patient-centered models of care, integrating biological, clinical, imaging, and digital data streams, may ultimately transform Fabry disease into a model of precision medicine for rare disorders. In this evolving framework, AI may emerge as the connecting bridge between biological complexity and clinical decision-making. A key challenge moving forward will be translating these technological advances into clinically validated, cost-effective, and widely accessible tools. This future-oriented connected-care ecosystem is schematically represented in Figure 6.

## 7. Conclusions

Fabry disease exemplifies the complexity of rare, multisystemic disorders in which a single genetic defect gives rise to a wide spectrum of clinical manifestations through intricate and interconnected pathogenic mechanisms. Over the past few decades, substantial progress has been made in elucidating the molecular basis of the disease, identifying key pathogenic pathways such as inflammation and oxidative stress, and developing disease-specific therapies. However, significant challenges remain, particularly in achieving early diagnosis, accurately predicting disease progression, and optimizing therapeutic strategies. In this context, the integration of biological, clinical, and computational approaches represents a paradigm shift in Fabry disease management. Artificial intelligence has the potential to bridge the gap between complex data and clinical decision-making, enabling more precise diagnosis, improved risk stratification, and personalized treatment. Ultimately, the transition from a traditional, reactive model of care to a proactive and predictive approach will be essential to improve long-term outcomes. This evolution will depend on the ability to integrate multimodal data, validate emerging technologies, and ensure equitable access to innovative diagnostic and therapeutic tools. Nevertheless, the successful implementation of these approaches will depend on rigorous validation and their integration into clinical workflows. Fabry disease thus represents a model for the broader application of precision medicine, in which molecular biology, precision diagnostics, advanced therapeutics, and artificial intelligence converge as complementary components to enable more precise and personalized clinical management and potentially provide a framework for other rare disorders.

## Figures and Tables

**Table 1:** **Major organ involvement in Fabry disease: molecular mechanisms, clinical manifestations, and representative biomarkers.** Overview of the multi-organ clinical spectrum of Fabry disease, highlighting the underlying pathogenic cascades and key diagnostic/monitoring tools. Abbreviations: Gb3, globotriaosylceramide; lyso-Gb3, globotriaosylsphingosine; hs-cTnT, high-sensitivity cardiac troponin T; NT-proBNP, N-terminal pro-B-type natriuretic peptide; MRI, magnetic resonance imaging; eGFR, estimated glomerular filtration rate.

Organ/System	Main Pathogenic Mechanisms	Major Clinical Manifestations	Representative Biomarkers/ Assessment Tools	References
**Cardiovascular** **system**	Gb3/lyso-Gb3-triggered cellular dysfunction in cardiomyocytes and endothelial cells, driving chronic inflammation, oxidative stress, microvascular impairment, and progressive fibrosis.	Left ventricular hypertrophy, myocardial fibrosis, arrhythmias, conduction abnormalities, heart failure	Lyso-Gb3, hs-cTnT, NT-proBNP, cardiac MRI with late gadolinium enhancement	[39,53,54]
**Kidney**	Podocyte injury; glomerular endothelial dysfunction; progressive fibrosis; inflammatory activation.	Microalbuminuria, proteinuria, chronic kidney disease, end-stage renal disease	Albuminuria/proteinuria, eGFR, lyso-Gb3, renal biopsy findings	[55,56,57,58]
**Peripheral nervous** **system**	Small-fiber dysfunction due to glycolipid accumulation; neuroinflammation; NF-κB activation.	Acroparesthesias, neuropathic pain, thermal sensory abnormalities	Quantitative sensory testing, skin biopsy, lyso-Gb3	[18,60,61]
**Central nervous** **system**	Endothelial dysfunction, microvascular injury, chronic inflammation, and altered cerebral perfusion.	Transient ischemic attacks, stroke, cognitive impairment, mood disorders	Brain MRI, inflammatory markers, neurocognitive assessment	[18,62,63,64,65,73]
**Skin**	Endothelial dysfunction and vascular ectasia secondary to glycosphingolipid accumulation.	Angiokeratomas, hypohidrosis, anhidrosis	Clinical examination, dermatological assessment	[59,66,67]
**Gastrointestinal tract**	Autonomic dysfunction, enteric nervous system involvement, vascular insufficiency.	Abdominal pain, diarrhea, nausea, early satiety	Clinical assessment, symptom questionnaires	[66,68]
**Auditory/Vestibular** **system**	Microvascular dysfunction, neuronal injury, inflammation.	Hearing loss, tinnitus, vertigo	Audiometry, vestibular testing	[63]

**Table 2:** Components of the precision diagnostic framework in Fabry disease.

Diagnostic Layer	Current Clinical Role	Main Limitations	Potential AI Contribution	References
**Clinical** **evaluation**	Recognition of suggestive multisystem manifestations and family history.	Phenotypic heterogeneity; diagnostic delay.	Pattern recognition from electronic health records and clinical phenotyping.	[74,76]
**α-Gal A enzymatic** **activity**	First-line biochemical screening in males.	Limited sensitivity in heterozygous females.	Integration with genetic and biomarker data.	[78]
***GLA* genetic** **testing**	Diagnostic confirmation; family screening; genotype–phenotype assessment.	Genetic variants of uncertain significance (GVUS).	AI-assisted variant classification and pathogenicity Prediction.	[79,89,90]
**Lyso-Gb3 and** **circulating** **biomarkers**	Diagnosis, disease severity assessment, therapeutic monitoring.	Not consistently elevated in late-onset disease and some females.	Multimodal biomarker integration and risk prediction.	[23,24,26]
**Cardiac and renal** **imaging**	Early detection of organ involvement and disease monitoring	Interpretation variability; operator dependence.	Automated image analysis and radiomics.	[82,83]
**Proteomics, metabolomics and lipidomics**	Discovery of disease signatures and novel biomarkers.	Limited clinical validation and standardization.	Feature extraction, biomarker discovery, patient stratification.	[84,85,86,87,88,91]
**Integrated multimodal diagnostics**	Precision diagnosis and personalized risk stratification.	Requires harmonized datasets and external validation.	AI-driven integration of clinical, imaging, molecular and omics data.	[27,89,90,92,93,94]

## Data Availability

No new data were created or analyzed in this study. Data sharing is not applicable to this article.
